# Nano‐delivery systems as a promising therapeutic potential for epilepsy: Current status and future perspectives

**DOI:** 10.1111/cns.14355

**Published:** 2023-07-14

**Authors:** Ahmad Movahedpour, Rasul Taghvaeefar, Ali‐Akbar Asadi‐Pooya, Yousof Karami, Ronia Tavasolian, Seyyed Hossein Khatami, Elahe Soltani Fard, Sina Taghvimi, Neda Karami, Khojaste Rahimi Jaberi, Mortaza Taheri‐Anganeh, Hassan Ghasemi

**Affiliations:** ^1^ Behbahan Faculty of Medical Sciences Behbahan Iran; ^2^ Epilepsy Research Center Shiraz University of Medical Sciences Shiraz Iran; ^3^ Department of Neurology, Jefferson Comprehensive Epilepsy Center Thomas Jefferson University Philadelphia Pennsylvania USA; ^4^ Department of Clinical Science, Faculty of Veterinary Medicine Shahid Bahonar University of Kerman Kerman Iran; ^5^ Department of Clinical Science and Nutrition University of Chester Chester UK; ^6^ Department of Clinical Biochemistry, School of Medicine Shahid Beheshti University of Medical Sciences Tehran Iran; ^7^ Department of Molecular Medicine, School of Advanced Technologies Shahrekord University of Medical Sciences Shahrekord Iran; ^8^ Department of Biology, Faculty of Science Shahid Chamran University of Ahvaz Ahvaz Iran; ^9^ TU Wien, Institute of Solid State Electronics Vienna Austria; ^10^ Department of Neuroscience, School of Advanced Medical Sciences and Technologies Shiraz University of Medical Sciences Shiraz Iran; ^11^ Cellular and Molecular Research Center, Cellular and Molecular Medicine Research Institute Urmia University of Medical Sciences Urmia Iran; ^12^ Abadan University of Medical Sciences Abadan Iran

**Keywords:** drug, epilepsy, exosomes, nanoparticles, seizure

## Abstract

Epilepsy is a common chronic neurological disorder caused by aberrant neuronal electrical activity. Antiseizure medications (ASMs) are the first line of treatment for people with epilepsy (PWE). However, their effectiveness may be limited by their inability to cross the blood–brain barrier (BBB), among many other potential underpinnings for drug resistance in epilepsy. Therefore, there is a need to overcome this issue and, hopefully, improve the effectiveness of ASMs. Recently, synthetic nanoparticle‐based drug delivery systems have received attention for improving the effectiveness of ASMs due to their ability to cross the BBB. Furthermore, exosomes have emerged as a promising generation of drug delivery systems because of their potential benefits over synthetic nanoparticles. In this narrative review, we focus on various synthetic nanoparticles that have been studied to deliver ASMs. Furthermore, the benefits and limitations of each nano‐delivery system have been discussed. Finally, we discuss exosomes as potentially promising delivery tools for treating epilepsy.

## Graphical abstract

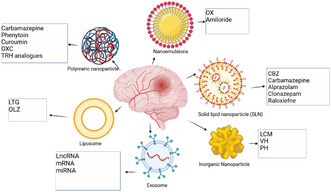

## 1 INTRODUCTION

**FIGURE 1:**
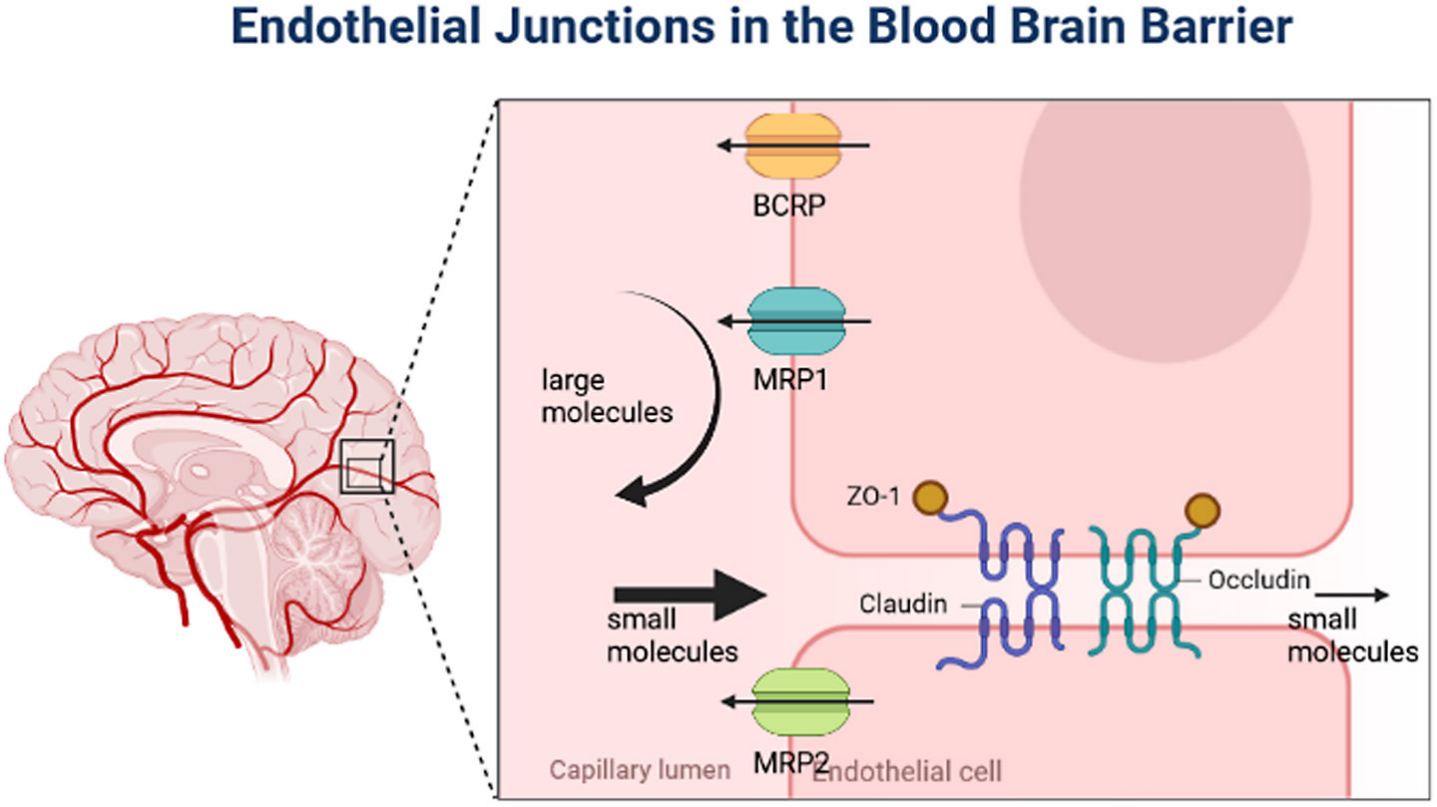
The BBB is composed of endothelial cells that are linked together by tight junction proteins. This barrier's tight junction proteins include claudin‐5, claudin‐1, and occludin. These tight connections keep harmful chemicals out of the blood and the brain. ZO‐1, ZO‐2, and ZO‐3 proteins link these tight junction proteins to the Actin cytoskeleton of endothelial cells. Different types of transporter proteins exclusively carry certain chemicals between the blood and the brain. BBB, blood–brain barrier; BCRP, breast cancer resistance protein; MRP, multidrug resistance protein; ZO, zonula occludens.

Epilepsy is one of the most common chronic neurological disorders that affects about 70 million people worldwide.^1^, ^2^ This brain disorder has various causes (e.g., genetic disorders, infections, traumatic brain injury, brain tumors, and metabolic abnormalities).^3^, ^4^ At the moment, there is no curative treatment for this disease, and the AEDs available on the market are only to control the symptoms and reduce the severity and frequency of seizures.^5^ Drugs such as lamotrigine or levetiracetam are major antiepileptic drugs that are administered systematically and can affect many organs other than the brain. For instance, sexual dysfunction and reproductive disorders are prevalent among male patients with epilepsy and can be a cause of systematic administration of AEDs such as carbamazepine. Therefore, rational drug therapy in epilepsy and, more importantly, targeted therapy can circumvent these downsides. However, various biological factors have complicated the treatment of this disease and reduced the effectiveness of existing therapies.^6^, ^7^ The blood–brain barrier (BBB) is one of the most important barriers to the effectiveness of ASMs.^8^ The endothelial cells lining the brain capillaries, along with other cells, including neurons, astrocytes, and pericytes, form a tight physical barrier that hampers the brain uptake of most unwanted substances from the blood (Figure 1).^9^, ^10^ There are several differences between the normal BBB and the BBB in epilepsy.

- **Increased permeability:** The BBB in epilepsy is more permeable than the normal BBB, allowing for the passage of larger molecules and cells that would normally be excluded from the brain. This increased permeability is thought to be due to a number of factors, including inflammation, oxidative stress, and changes in the tight junctions that line the cerebral capillaries.^11^
- **Altered expression of transporters:** The BBB in epilepsy also has altered expression of transporters, which are proteins that help regulate molecules' movement across the BBB. This altered expression can lead to imbalances in the levels of certain nutrients and chemicals in the brain, which can contribute to seizures.^12^
- **Neuroinflammation:** Neuroinflammation is a common feature of epilepsy and can also contribute to BBB dysfunction. Neuroinflammation is caused by the activation of microglia, which are immune cells that reside in the brain. Microglia can release a variety of inflammatory factors that can damage the BBB and increase its permeability.^13^

To treat CNS disorders such as epilepsy, medications must cross the BBB and access the brain tissue. However, it has been shown that only low molecular weight (<1000 Da) lipophilic molecules can cross the BBB. Furthermore, it has been estimated that due to the BBB, 100% of large molecules and virtually 98% of small molecules cannot cross the blood–brain barrier satisfactorily for therapeutic effects, and this can limit the effectiveness of drugs for the treatment of epilepsy.^3^, ^14^, ^15^ There are also several efflux pumps, such as Breast Cancer Resistance Protein (BCRP), Multidrug resistance‐protein (MRP1), and (MRP2), in the luminal membrane of endothelial cells, which restricts the access of drugs to the brain and prevents them from staying there, thus leading to the development of drug resistance.^3^ Drug resistance is an important issue in the treatment of epilepsy and occurs in 20%–25% of patients. Although various mechanisms have been reported for this process, the transporter hypothesis has attracted much attention. According to this hypothesis, AEDs can act as substrates for the above‐mentioned transporters.^14^ Therefore, due to the high incidence of epilepsy and inefficient treatment of these patients, it is necessary to consider solutions to facilitate AEDs entry and persistence in the brain to improve their effectiveness.

**FIGURE 2:**
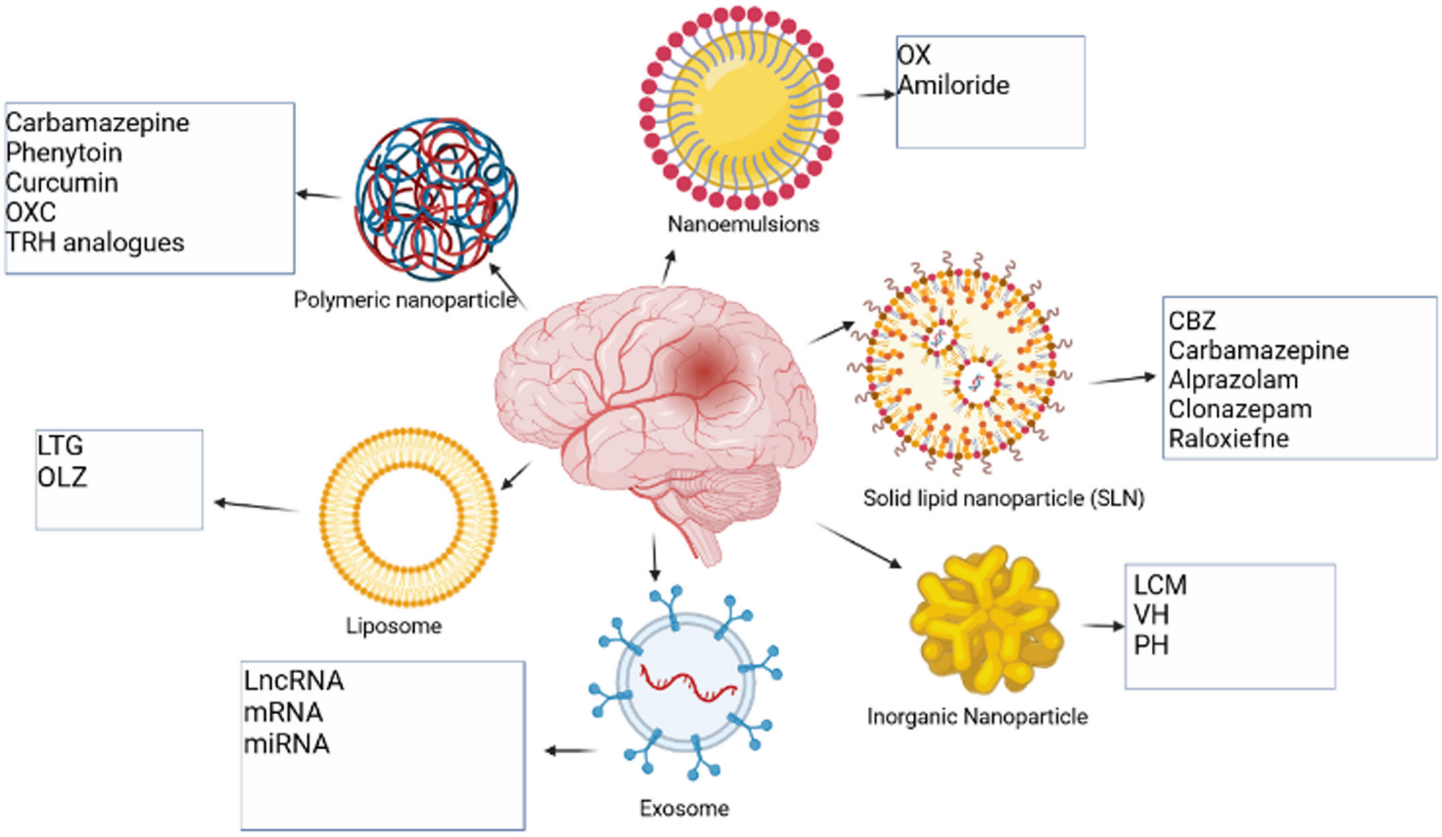
Nanotechnology‐based medication delivery devices are being used to treat epilepsy. As discussed in this review, intelligent NP designs that optimize delivery have the potential to increase precision medicine performance and thereby hasten clinical translation. Each type has distinct benefits and disadvantages in terms of cargo, delivery, and patient reaction. CBZ, Carbamazepine; LCM, lacosamide; lncRNA, long non‐coding RNA; LTG, Lamotrigine; miRNA, microRNA; mRNA, messenger RNA; OLZ, olanzapine; OX, oxcarbazepine; OXC, oxcarbazepine; TRH, thyrotropin‐releasing hormone; VH, valproic acid.

**FIGURE 3:**
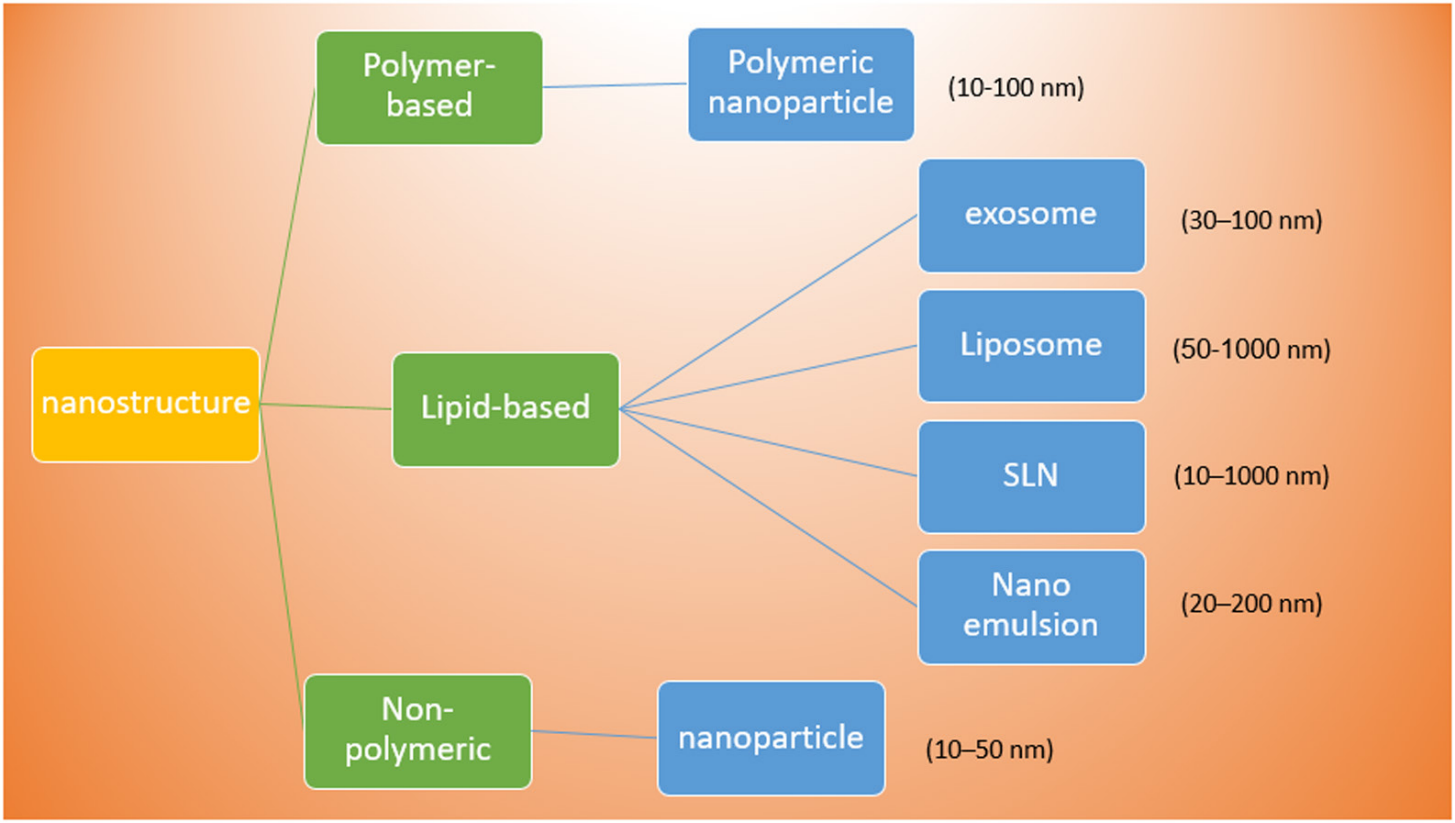
Classification of nanomaterials.

Recently, nanotechnology has opened a potential horizon in the treatment of epilepsy (Figure 2). Various synthetic nano‐carriers, like liposomes, polymeric nanoparticles, and inorganic nanoparticles, have been used to deliver ASMs (Figure 3) (Table 1).^16^ Similarly, exosomes, as natural nano‐carriers, have attracted the attention of researchers because of their potential in targeted drug delivery.^17^ In this narrative review, we discuss different types of synthetic nano‐carriers that have been studied for the delivery of ASMs (Table 2). In addition, we also discuss the potential benefits of exosomes in the delivery of ASMs. This review may pave the road for future research in the field.

**TABLE 1:** Various types of nanoparticles.

Nanosystems	Advantages	Disadvantages	References
Liposomes	This encapsulates both hydrophilic and lipophilic drugs, in their various phases Keeping drugs safe from extreme environmental conditions In addition, biodegradability and biocompatibility are of superior quality Low toxicity Longer duration of circulation	Poor stability as a result of phospholipids and their predisposition to oxidative degradation requiring special storage	[72]
Polymeric nanoparticles	The release of drugs occurs in a controlled and sustained manner Encapsulating drugs that are hydrophilic and hydrophobic Physical and chemical properties that are tunable When desired, a large number of biodegradable materials can be used The synthesis of preferred polymers requires consideration of various properties such as pH, enzymes, hydrolysis, and others: Data reproducibility when using synthetic polymers High stability compared to lipid‐based ones It is possible to prepare them in a variety of ways	Difficulty in scaled up production Insufficient toxicological assessments in the literature	[72]
SLNs	Tunable and small size Stability Easily functionalize surfaces During fabrication, organic solvents are not used High‐scale production Sustaining and controlling the release Hydrophilic and hydrophobic drug delivery No toxicity Low immunogenicity Biodegradation	The loading capacity is low Expulsion of drugs during storage	[40]
NEs	Molecules protection; absence of toxicity as a result of using biocompatible and biodegradable materials	Poor stability during storage and the gradual release of the encapsulated molecules (therefore must be produced right before use)	[46]
Inorganic nanoparticles	Small size Multi‐functionality Theranostic use	Low biocompatibility If the RES are not functionalized, they can be cleared quickly	[40]
Exosomes	Low toxicity Low immunogenicity Inherent tissue tropism Therapeutic effects of inherent exosome contents Ability to bypass BBB Good biocompatibility and stability Capacity to be packed with a variety of therapeutic cargos	Lack of standardized protocol for isolation, purification and mass production Limited drug loading efficiency Unfavorable effects of inherent exosome contents	[66, 68, 70, 71]

**TABLE 2:** A brief summary of the studied drug delivery systems in epilepsy.

Nanoparticles (Class)	Drugs	Outcomes	References
Inorganic nanoparticles (gold nanoparticles)	Lacosamide (LCM)	In the presence of glucose‐coated gold nanoparticles that are conjugated to lacosamide, seizures are reduced in severity and frequency. Gold nanoparticles coated with glucose are promising nanocarriers capable of transferring antiepileptic drugs efficiently to brains in patients with drug‐resistant epilepsies. Novel treatment modalities for refractory epilepsy may benefit from targeting BBBs for drug delivery.	[73]
Lipid nanoparticle (SLN)	Carbamazepine (CBZ)	After treatment of the SLN with chitosan, they were able to obtain better anticonvulsant activity using the MES method. SLN without chitosan performed better with the INH method.	[74]
Lipid nanoparticle (NLC)	Valproic acid (VPA)	When administered through intranasal routes, VPA‐NLCs resulted in improved BA when compared to IP.	[75]
Lipid nanoparticle (NLC)	Lamotrigine (LTG)	A higher concentration of LTG in brain can be achieved by administering LTG‐NLCs as opposed to orally ingesting LTG.	[76]
Lipid nanoparticle (NLC and SLN)	Carbamazepine	A thermosensitive mucoadhesive gel was incorporated into the formulation. The NLC significantly reduced the effects of chemically induced convulsions in the animals.	[77]
Inorganic nanoparticles (microporous silica nanoparticles)	Valproic acid and phenytoin (PHT)	Inflammation or necrosis was not caused by the implants. Neurons close to the reservoir showed no pathological effects or damage on stained sections.	[78]
Polymeric nanoparticle	Carbamazepine	30 times more effective than the free drug. The encapsulated CBZ is unaffected by the PgP porter.	[15]
Polymeric nanoparticle (PBCA)	Phenytoin	In rats resistant to PHT, anticonvulsant activity was observed and the AUC ratio of [PHT] was higher than when PHT was administered.	[79]
Nanoliposome	Lamotrigine	LTG nanooliposomes delivered into goat nasal mucosa penetrated better than the suspension, and a nasal toxicity study indicated it was a safe formulation for delivery into the brain by nasal route.	[80]
Lipid nanoparticle (SLN and NLC)	Clonazepam	Gel formulations were incorporated into thermosensitive mucoadhesive gels. Chemically induced convulsions had been found to be considerably controlled when glyceryl monooleate NLC was administered to the animals.	[81]
Lipid nanoparticle (NLC)	Carbamazepine	CBZ aqueous solubility increases resulting in improved brain delivery.	[82]
Lipid nanoparticle (SLN)	Diazepam	Good encapsulation efficiency and significant and prolonged release observed.	[83]
Polymeric nanoparticle	Diazepam	Diazepam can be encapsulated as an ASM using NP.	[84]
Nanoemulsion	Oxcarbazepine (OXC)	The MTT assay showed that encapsulating the drug in emulsomes decreased its toxicity. OXC can be incorporated into emulsomes to produce stable nanoformulations. Adapting the surface charge and particle size of emulsomes to modulate their properties created an emulsion that had a prolonged release profile and residence time, and demonstrated direct norepinephrine to brain transport in rats.	[85]
Polymeric nanoparticle (PLGA)	Oxcarbazepine	Neuroprotection; reduction in the number of times the drug must be administered by comparing it with the free drug.	[86]
Lipid nanoparticle (NLC)	Lamotrigine	Compared to IN and oral administration, the drug spends more time in the brain. In a lower dose, IN administration has a greater protective effect than oral administration.	[76]
Polymeric nanoparticle (PLGA)	Carbamazepine	With the Pgp inhibitor verapamil, CBZ exhibits a greater anticonvulsant effect and a reduced effective dose; CBZ‐NPs cause an anticonvulsant effect that's 30‐fold greater.	[15]
Polymeric nanoparticles	Oxcarbazepine	Effects on the nervous system. Keeping the anticonvulsant activity while reducing the dosage regimen. Induction of accumulation in the cerebral tissue model. Neuronal compatibility with this novel system of drug administration.	[86]
Lipid nanoparticle (NLC)	Valproic acid	Intranasal administration leads to higher brain concentration. Using lower doses of soy lecithin octyldodecanol at the same concentrations as systemic administration provides the same protection.	[75]

## 2 TRANSPORT PATHWAYS ACROSS BBB

BBB transcellular transport involves three main mechanisms: active efflux transport (AET), receptor‐mediated transport (RMT), and carrier‐mediated transport (CMT). RMT is a specific type of endocytosis that enables the non‐invasive transport of macromolecules, such as antitumor proteins, across BBB. Commonly used receptors include insulin‐like growth factor 1 receptor, low‐density lipoprotein receptor‐related protein 1 (LRP1), and transferrin receptor (TfR). CMT is facilitated by a range of solute carrier (SLC) transporters that convey substances such as sugar, amino acids, organic cations or anions, and nutrients into the brain.^18^ Key SLCs include glucose transporters (GLUTs), monocarboxylate transporters, organic ion transporters (both cationic and anionic), and nucleoside transporters. AET is an ATP‐driven process that serves as the primary transcellular transport pathway. It prevents foreign substances (including potentially toxic substances and therapeutic drugs) into the brain and transports compounds that have crossed the BBB back into circulation, thereby playing a detoxifying role. The primary drug efflux transporter is the ATP‐binding cassette (ABC) transporter superfamily. These three transport mechanisms can be fully leveraged to increase the transport of therapeutic drugs by their corresponding transporters, thereby enhancing drug crossing of the BBB and improving therapeutic outcomes.^19^

## 3 SYNTHETIC NANO‐DELIVERY SYSTEMS FOR EPILEPSY

Synthetic nano‐delivery systems are developed for the target‐specific delivery of various drugs.^20^ These systems may lead to more effective treatment of various disorders (e.g., by improving direct drug delivery and also optimizing drug release patterns).^5^, ^20^ Various synthetic nano‐delivery systems are currently under investigation (e.g., liposomes, polymeric nanoparticles, solid lipid nanoparticles, nano‐emulsions, and inorganic nanoparticles).^5^

### 3.1 Liposomes

One lipid bilayer structure used as a good method for drug delivery is the liposome; its structure is similar to the cell membrane. Liposomes may transport Different substances into the cells, but the results are usually not as expected in clinical trials. Despite all the limitations, liposomes are used in clinical trials.^21^

### 3.2 Polymeric nanoparticles

One of the most widely used controlled drug delivery systems (CDDS) is polymeric nanoparticles (PNP) because of their numerous benefits.^22^ PNPs have a typical size of 10–100 nm, and their constituent matrices are either natural (e.g., chitosan or gelatin) or synthetic (e.g., polycaprolactone). Biodegradable or non‐biodegradable polymers can be used for making them (such as cyanoacrylate or poly(lactic‐co‐glycolic acid) [PLGA]) (e.g., polyurethane).^23^, ^24^

The surface charge of polymeric nanoparticles can be either positive or negative depending on the polymer composition, which determines their biological characteristics. Bio‐adhesiveness, cell penetration, and muco‐adhesiveness are also based on this feature.^25^ PNPs can also be biologically active and transportable, depending on their structure. PNPs could be prepared as nanocapsules or nanospheres, yielding the drug encasing or enveloping the polymer matrix.^26^ A notable benefit of nonionic surfactants is their potential to reduce interactions with phagocytic systems by opsonizing them, for instance, which reduces phagocytosis.^24^

PNPs are the potential alternative for improved drug delivery systems in the treatment of ND^27^ and offer various advantages, such as (i) being supplied through a systemic route of administration and intended to reach any human organ because of their nano‐metric size and (ii) providing a controlled release manner from the matrix structure into a targeted part of the body (iii) protect drugs from enzymatic degradation, therefore, provide good in vitro and in vivo stability (iv) possibility to change surfaces with ligands (v) solubilize large amounts of lipophilic drugs (vi) their preparation methodologies are cost‐effective and easily scalable that will yield targeted delivery of drugs to the BBB—increasing the pharmacological activity of the drugs in the CNS and reducing the side effects as well as the frequency of dosages to improve the patient compliance.^28^, ^29^, ^30^, ^31^

PNPs possess a great deal of potential whenever it comes to drug delivery to the CNS. Another advantage of PNPs is their targeted delivery, achieved by surface modification, and enables directing these CDD systems toward the brain.^32^ PNPs are able to deliver not only small molecule therapeutics but also nucleic acids (e.g., DNA, RNA),^33^ proteins,^34^ and diagnostic compounds to the brain to prevent degradation.^35^

### 3.3 Solid‐lipid nanoparticles (SLNs)

Lipid nanoparticles are categorized into two types, solid lipid nanoparticles (SLN) and nanostructured lipid carriers (NLC), which are the second generation of LNs. Emulsions, liposomes, and polymeric micro and nanoparticles are traditional colloidal carriers that have been replaced by SLNs, which were first introduced in 1991.^36^, ^37^ SLNs are biocompatible lipid‐based nanocarrier systems composed mostly of lipid or modified lipid nanostructures (triglycerides, fatty acids, or waxes) with submicron diameters less than 1000 nm.^38^ It should be noted that one of the major reasons for the wide adoption of SLNs has been their capability to deliver both lipophilic and hydrophilic drugs, as well as gene, oligonucleotide, peptide, and even smaller nanoparticles such as superparamagnetic iron oxide nanoparticles, to a wide range of diseased tissues.^39^ As well as reducing toxicities and protecting therapeutic molecules, SLNs also transfer molecules from the reticuloendothelial system (RES). Due to their low water solubility, they provide controlled and sustained release of encapsulated chemicals; in addition, SLNs can be used for longer periods of time due to their increased long‐term stability.^40^ These lipid carriers have a number of advantages, such as the protection of drugs against extreme environmental conditions, facile scaled‐up synthesis using high‐pressure homogenization, biocompatibility, and, lastly, biodegradability.^41^ SLNs are synthesized from a variety of surfactants and/or co‐surfactants and a range of lipids with similar properties, such as low melting point and solidity at room and body temperatures. Moreover, SLNs are biocompatible, can be sterilized in a straightforward manner, and organic solvents are not required in their fabrication methods that can positively affect the toxicity of the final product. Two final advantages of lipid nanocarriers are their facile scaled‐up manufacturing that is amenable for industrial purposes. SLNs can be functionalized with specifically targeted targeting lipids, which allows them to be targeted to specific tissues.^40^, ^42^

SLNs are known as one of the safest and cheapest drug carriers that provide non‐toxic, effective, and safe treatment for neurological disorders by crossing the BBB. To portray the functionality and efficacy of SLNs, we need to shed light on the modern fabrication technologies for the production of SLNs as drug carriers; this is largely due to the fact that their efficacy and functionality are dependent on their constituents, size, structure, physico‐chemical properties, and synthetic procedures. Newly synthesized lipid nanoparticles have progressively improved the applicability and advantages of SLNs as drug carriers.^38^, ^43^

### 3.4 Nano‐emulsions (NEs)

Nanoemulsions are formed due to the combination of two immiscible liquids and are known as one of the best drug delivery systems with kinetic stability and improved solubility. As the name implies, Nano has droplet sizes ranging from 20 to 200 nm.^44^

Because of their ability to solubilize non‐polar active chemicals, NEs have been proposed for various pharmacy applications as drug delivery systems. However, as nanoemulsions suffer from stability problems, they are produced right before use, and a majority of suggested formulations are self‐emulsifying systems.^45^ NEs are made up of extremely small emulsion droplets, which are usually oil droplets in water. NEs, like normal emulsions (with diameters > m), are in a non‐equilibrium state from a thermodynamic point. A stable nanoemulsion comprises three key components: aqueous phase, oil phase, and surfactant.^45^, ^46^

NEs share some of the same advantages as other lipid drug carriers; including increased efficiency in molecule encapsulation; facile scaling up production techniques; protection of molecules in the face of adverse environmental conditions; absence of toxicity due to the use of biocompatible, biodegradable, and approved pharmaceutical ingredients; and their potential to be utilized in various administration routes. However, several publications have found low stability and release of the encapsulated molecules after storage.^47^

NEs are utilized as drug delivery systems for a variety of systemic routes of administration. Parenteral (or injectable) nanoemulsion administration is used for a variety of purposes, including nutrition (e.g., fats, carbohydrates, vitamins, etc.), controlled drug release, and targeting of pharmaceuticals to specific parts of the body, vaccine delivery, and gene carriers.^48^

### 3.5 Inorganic nanoparticles

Inorganic nanoparticles such as gold, iron oxide, silica, and silver are under investigation for preclinical and clinical trials in treating, diagnosing, and detecting various disorders.^49^, ^50^, ^51^ Furthermore, many of the inorganic substances used to produce nanoparticles have long been employed in the clinic for a wide range of therapeutic purposes.^52^, ^53^ Two well‐known examples of inorganic compounds with applicability in therapeutics are platinum (e.g., cisplatin, carboplatin, oxaliplatin, etc.) which is widely used in cancer treatment, and silver ions which are often exploited as an antibacterial agent.^54^, ^55^ Inorganic nanoparticles offer unique opportunities for clinical diagnostic and therapeutic techniques that polymeric and other traditionally used nanoparticles do not provide. For example, Ex vivo detection with inorganic nanoparticles is presently explored as an ongoing clinical trial to identify stomach lesions in patient breath using AuNP and carbon nanotube functionalized biosensors.^56^

Inorganic nanoparticles have considerable advantages in the biomedical field due to their large surface area, tunable structures, various surface chemistry, and unique optical and physical properties. As a result, inorganic nanoparticles and their metal ions have been exploited as therapeutic agents targeted to specific tissues or in the treatment of various diseases with no detectable acute toxicity by researchers worldwide.^40^

These nanoparticles' exploitation in treating brain diseases is quite novel. NPs enable the effective loading of therapeutic compounds due to their high surface‐to‐volume ratio and provide alternative therapeutic options due to their material‐distinct intrinsic properties.^57^, ^58^

Cisplatin tethered gold nanoparticles are used as a treatment regimen in glioblastoma multiform as both drug transporters and radiosensitizers in radiotherapy—emitting ionizing photoelectrons and Auger electrons.^59^

Likewise, iron oxide nanoparticles in an oscillating magnetic field generate heat which can (i) be used as a hyperthermia treatment method in glioblastoma patients,^60^ (ii) temporally disrupt the blood–brain barrier to enable nanoparticles delivery to the cerebral tissue,^61^ and (iii) promoting NP uptake by opening heat‐sensitive ion channels.^62^, ^63^ However, effectively employing NPs as drug delivery systems or therapeutic agents requires a more profound understanding of the principles controlling their interaction and functional effects in neuronal circuits.^64^

### 3.6 Exosomes as naturally nano‐carriers for epilepsy

Extracellular vesicles (EVs) are biological particles enclosed by a lipid bilayer membrane that are secreted by almost all cells and are present in different bio‐fluids like blood, semen, urine, saliva, breast milk, and cerebrospinal fluid.^65^ Based on their size and biogenesis mechanism, EVs are classified into three categories: Apoptotic bodies, micro‐vesicles, and exosomes. Unlike micro‐vesicles and apoptotic bodies derived from plasma membranes and apoptotic cells, respectively, the biogenesis process of exosomes as the smallest EVs (30–100 nm in diameter), starts from endosomes.^66^ Initially, the clathrin‐coated areas of the plasma membrane sprout inward to form endosomes. Then, the multivesicular bodies (MVBs) are formed following the inward budding of the membrane of the endosome. MVBs have two general destinies: they can attach to the lysosome and be degraded, or they can be fused to the cell membrane and release their intraluminal vesicles (ILVs), called exosomes, into the extracellular space.^65^ It is interesting to note that exosomes were initially conceived as cellular wastes, but further research revealed that depending on their cellular origin, exosomes are packed with different functional molecules like messenger RNA (mRNA), DNA, long non‐coding RNA (LncRNA), and micro RNAs (miRNAs) that can be entered to recipient cells and impress their behavior and characteristics.^67^ Therefore, exosomes act as natural nano‐carriers in the body and can be inspired to deliver various medications, including ASMs. Delivery of drugs by exosomes has many advantages^66^, ^68^: (i) Compared to synthetic nanoparticles, due to their endogenous origin, exosomes have less toxicity and are less likely to be cleared by macrophages and reticuloendothelial cells, so they have a longer half‐life in the bloodstream. (ii) The BBB is permeable to exosomes, and they can easily bypass it and encounter brain tissue. Accordingly, they may be suitable carriers for the delivery of therapeutic agents to the brain to treat epilepsy. (iii) Exosomes released from different cellular sources carry a variety of membrane ligands that facilitate their targeting of specific tissues and their effective cellular uptake. (iv) Exosomes have a variety of biological substances in their lumen that can be therapeutically useful. For example, Long et al.^69^ showed in a study that intranasal administration of human bone marrow‐derived mesenchymal stem cells released exosomes led to normal neurogenesis maintenance and reduction in inflammation and neuron loss in animals after status epilepticus. To our knowledge, although no study has yet been published on the delivery of ASMs by exosomes, it seems that they can compete with synthetic nanoparticles due to their better safety, selectivity, and circulation half‐life. Besides, their ability to cross the BBB is an important feature.

Nonetheless, as shown in Table 1, improving the exosome isolation and purification methods is an important issue that should be taken into consideration for their clinical use. Furthermore, despite the existence of numerous techniques (e.g., incubation, sonication, electroporation, etc.) for drug loading into the exosomes, limited loading efficiency is another challenge that needs to be resolved.^66^, ^68^, ^70^ It should also be borne in mind that the internal content of exosomes inherited from their parent cells acts as a double‐edged sword and, along with beneficial effects, they can have unfavorable effects, which highlights the importance of the careful selection of the exosome‐producing cell source for drug delivery.^71^

## 4 CONCLUSION

Successful treatment of epilepsy requires new approaches. Nanotechnology may prove to be more effective in treating PWE than traditional treatments with ASMs. In addition, the designed nanomaterials' exceptional properties may offer them superior benefits than the existing ASMs (e.g., increased biocompatibility, increased blood circulation time, reduced systemic toxicity, etc.). Therefore, it is essential to design and conduct preclinical studies to clarify the role of drug delivery systems with nanomaterials in epilepsy.

## FUNDING INFORMATION

This study was financially supported by Abadan University of Medical Sciences, Abadan, Iran (Grant number: 1401U‐1465).

## CONFLICT OF INTEREST STATEMENT

The authors declare no conflicts of interest.

## Data Availability

Data sharing is not applicable to this article as no new data were created or analyzed in this study.
